# Satellite DNAs rising from the transposon graveyards

**DOI:** 10.1093/dnares/dsaf026

**Published:** 2025-09-30

**Authors:** Eva Šatović-Vukšić, Patrik Majcen, Miroslav Plohl

**Affiliations:** Division of Molecular Biology, Ruđer Bošković Institute, 10000 Zagreb, Croatia; Division of Molecular Biology, Ruđer Bošković Institute, 10000 Zagreb, Croatia; Elementary School Trnovec, 42202 Trnovec, Croatia; Division of Molecular Biology, Ruđer Bošković Institute, 10000 Zagreb, Croatia

**Keywords:** satellite DNAs, transposable elements, heterochromatin, genome evolution

## Abstract

Repetitive DNA sequences, as transposable elements (TEs) and satellite DNA (satDNA) spread and diversify within host genomes, impacting genome biology in numerous ways. In the first part of this review, we emphasize the evolutionary importance of satDNAs and TEs, providing a short summary of their roles and the mechanisms by which they influence the structure and function of genomes. We also discuss the broad, complex, and extensive relationships between TEs and satDNAs. Following that, we bring together different mechanisms on the generation of satDNA from TE, as it has been demonstrated that almost any part of any type of TE can undergo tandemization and produce novel satDNAs. Importantly, we here present a hypothesis that would explain the existence of particular types of monomers, namely composite satDNA monomers which display multiple subsequent stretches of similarity to various TEs, for which the explanation was lacking so far. We propose that even highly shuffled and degraded TE remnants residing in heterochromatin ‘TE graveyards’ can give rise to new satDNA sequence monomers, transforming these genomic loci into DNA ‘recycling yards’. Furthermore, we emphasize important evolutionary questions regarding the causes, mechanisms, and frequency of these occurrences.

## 1. Introduction

Repetitive DNA components build a significant portion of eukaryotic genomes. They are traditionally divided into 2 major groups, satellite DNAs (satDNAs), comprised of arrays of sequences repeated in tandem, and transposable elements (TEs), interspersed throughout the genome.^1–5^ According to conventional concepts, satDNAs are dominant in the pericentromeric, subtelomeric, and interstitial chromosomal regions, where they constitute blocks of constitutive heterochromatin.^6^ The most basic classification of TEs divides them into 2 main groups according to their transposition intermediates: Class I (Retrotransposons), and Class II (DNA transposons). In the ‘copy-and-paste’ mechanism employed by Class I elements, an RNA intermediate is present. The majority of Class II elements are mobilized by ‘cut-and-paste’ process, in which the transposon is excised and transferred to a new genomic site.^7^

SatDNAs and TEs are often called the ‘dark matter’ of the genome, as their functions were initially unknown. In addition, the repetitive nature of these sequences causes significant technical problems in sequencing and assembly, resulting in their general underrepresentation in outputs of genome projects.^8,9^ New sequencing methodologies, in particular long-read sequencing supported by specialized bioinformatics tools, are on the way to solve these problems and to shed more light on the genomic composition of the ‘dark matter’ repeats.^8,10^ As a result, scientific literature has extensively covered numerous topics related to the structure, organization, function, and evolution of these sequences in diverse model and non-model organisms, enabling new insights into repetitive DNAs.^6,11–19^ Both satDNAs and TEs have tremendous impact on genome architecture and evolution, making them crucial players in the process of comprehending the overall structure and function of the genome.

In this article, we bring the basic notions on satDNAs and TEs, summarize their evolutionary significance and their crucial contributions to the constitution and evolution of eukaryotic genomes. We discuss the intricate and extensive connections between TEs and satDNAs, as new data are enhancing our understanding of these relationships. We summarize various mechanisms regarding the generation of satDNAs from TEs. Notably, we present a hypothesis that seeks to explain the existence of satDNA sequences that share short stretches of similarity with multiple TEs, suggesting that these satDNA sequences may originate from ‘TE graveyards’.

## 2. Evolutionary significance of satDNAs and TEs

**Fig. 1.**
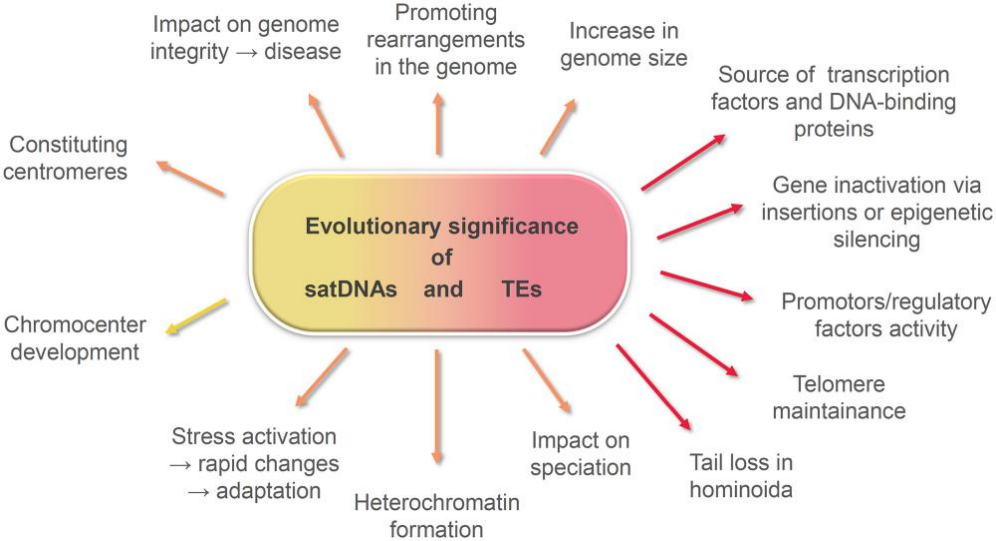
Schematic depiction of the major contributions of satDNAs (yellow) and TEs (red) to genome structure, function, and evolution. Shared roles are denoted by orange arrows.

TEs and satDNAs play crucial roles in various processes that significantly influence the structure and function of the genome. While TEs and satDNAs differ in their structures, mechanisms of dissemination, and organizational patterns, there are numerous connections between them, which will be explored in the following sections. On one hand, they often complement each other in some of the genomic functions (Fig. 1). On the other hand, due to substantial differences in their structure and organization, many of the roles that TEs and satDNAs fulfil in the genome are specific to certain subclasses of these repetitive elements.

As the main common role, TEs and satDNAs shape the overall genomic landscape by inducing structural rearrangements through illegitimate recombinations, deletions, inversions, translocations, and segmental duplications. Both TEs and satDNAs can make significant changes to the genome size.^20–25^

The domestication of TEs has yielded numerous proteins, which are co-opted for functions in physiology of many species. For instance, numerous DNA-binding proteins and transcription factors are derived from transposases, and the majority of primate-specific regulatory sequences are derived from TEs (7). In addition, TE machinery serves as a mediator in the formation of intron-free gene copies (retrogenes), which can lead to the evolution of a novel trait through neofunctionalization.^26^ TE can also serve as telomeric sequences at the ends of chromosomes, such as in *Drosophila.*^27^

TEs extensively affect regulatory networks that are involved in processes like dosage compensation, immunity, and early embryonic development. There are numerous examples of changes in phenotype driven by TE activity, some of them being beneficial for the organism.^12^ The colour polymorphism of the peppered moth is a well-known case of such adaptive, TE-induced change.^28^ Recently, their involvement in the mechanism that facilitated tail-loss process in hominoids has been proposed.^29^ In addition to the direct gene inactivation, TE insertions can also affect the expression of nearby genes by alterations at the epigenetic level (eg histone modifications and chromatin packing).^30^ TE Helitrons were shown to be powerful genome shuffling agents with wide-reaching biological consequences.^31^ TE movement, regulatory activities, and effects on genome integrity can also cause (and intensify) the effects of many diseases.^7^ In addition, differences in TEs distribution can influence speciation through the formation of reproductive isolation.^32^

Centromeres are essential for proper segregation of chromosomes, and satDNAs and TEs are the most common DNA component in centromeres of animals and plants.^33^ Typically, satDNAs and TEs extend to the pericentromeric regions much more than it would be necessary for the centromeric function alone. Pericentromeric satDNA repeats were shown to be the main contributors to large-scale nuclear organization that supports general transcription.^34^ In addition, satDNA sequences have been continuously associated to evolutionary breakpoint areas and fragile sites in a variety of taxa.^13^ There are numerous neurological, congenital, and developmental disorders caused by short tandem repeat expansions,^35^ and changes in the repeat copy number can also affect social bahaviour.^36^

SatDNAs have key roles in heterochromatin formation and maintenance,^37^ and contribute to heterochromatin organisation in embryonic stem cells.^38^ TEs were also suggested to be mediators and facilitators of heterochromatin formation, through recruitment of Heterochromatin Protein 1 and repressive chromatin marks.^39^ Different studies revealed that satDNA transcription and satDNA transcripts are involved in various cellular processes, while the improper regulation of satDNA transcription was shown to be associated with genomic instability and human diseases.^13,40^ Pericentromeric satDNA transcription is significantly elevated across many cancers, and these transcripts have a variety of biological functions in either aiding the cancerous state (mutation induction, disruption of epigenetic regulation, tumour cell proliferation, inflammation, resistance to cancer treatment, destabilizing genome integrity), or opposing it (innate immune system activation). Elevated transcription of pericentromeric satDNAs is typically triggered by various environmental stressors, initializing the mechanism that can affect regulation of many genes through reversible changes in the chromatin state.^40^ Under stress conditions, TEs are also activated. This activation may modify genes structure and activity of genes, aiding in the process of adaptation and survival.^41,42^

SatDNAs are involved in other numerous processes that are crucial for the cells. For example, it has been demonstrated that the formation of the chromocenter and the maintenance of the entire genome within the nucleus rely on the presence of satDNA repeats located across multiple chromosomes. The chromocenter contains DNA-binding proteins and physically links different chromosomes by bringing together their corresponding pericentromeric satDNA repeats.^43^

## 3. Relationships between satDNAs and TEs

An increasing number of reports shows that satDNAs and TEs are interconnected in various ways (reviewed in Šatović-Vukšić and Plohl^16^ and Zattera and Bruschi^44^). The heterochromatin is the most frequent site of interaction, as both types of repetitive sequences are particularly prevalent in this genomic region. There, they not only coexist but also form complex and dynamic networks. It was observed that 1 satDNA array can be directly followed by satDNA of another type, and different types of TEs can be simultaneously found in the immediate vicinity of a satDNA. TEs can be inserted into satDNA arrays or inserted into other TEs.^45^ Moreover, cases of multiple insertions have been reported, such as a TE being inserted within another TE, both of which are located within a satDNA array.^46,47^

Neither satDNA nor TEs are exclusively limited to heterochromatin.^16,48^ SatDNA sequences outside of the heterochromatin can be found in different organizational forms: as single monomers or monomer fragments, in arrays of various (usually short) length, or incorporated into TEs.^45,49–55^

TEs significantly contribute to satDNA evolution by generating repeats that can be dispersed through the genome, and in some cases, amplified into long arrays of novel satDNAs. SatDNA repeats of various species were formed by tandemization of a complete TE or its subsegments and structural components.^56–60^ This way, satDNA can arise from the sequence segment of a long-terminal repeat (LTR), gag or pol domains of LTR retrotransposons; untranslated regions of LINE; terminal inverted repeats or sequence segments from the central parts of DNA transposons.^44,61^ Additionally, satDNAs can be formed through the expansion of short-internal arrays found within TEs.^50,52,62,63^ A common example of such expansions is observed in Helitron/Helentron TEs, which have conserved sequence segments at their ends, while their central regions often contain arrays of satDNA-like repeats.

TEs are also proposed to facilitate and contribute to genomic dispersal of satDNA repeats.^45,51,62,64–66^ In line with that, it was shown that TEs are responsible for novel, highly dispersed organization of numerous satDNAs across the whole-genome, completely contrasting canonical concepts of compartment-localized satDNA organization.^54^ In extreme cases, complete satelitomes (entirety of satDNAs in 1 organism), comprised of numerous satDNAs, can be based on TEs (being TE-derived, TE-incorporated, or TE-propagated).^55^

Hybrid forms between satDNAs and TEs also exist. For example, ‘transitional’ 154TR sequence is at the same time a tandem repeat embedded in a TE, but also found as large expanded arrays within constitutive heterochromatic loci, similar to classical satDNAs.^67^ The Cg170/HindIII sequence displays fluctuation between 3 forms, TE-incorporated, standalone satDNA arrays, and an ‘intermediate’ form. In the case of the latter, tandem repeats were found to be associated with TE Helitron components only on one side of the array.^55^ These intermediate arrays may result from recombination events between element-incorporated and standalone arrays.^54^ Alternatively, they may be generated by aberrant rolling circle replication (RCR), as will be discussed later.

## 4. Mechanisms that generate satDNAs (from TEs)

**Fig. 2.**
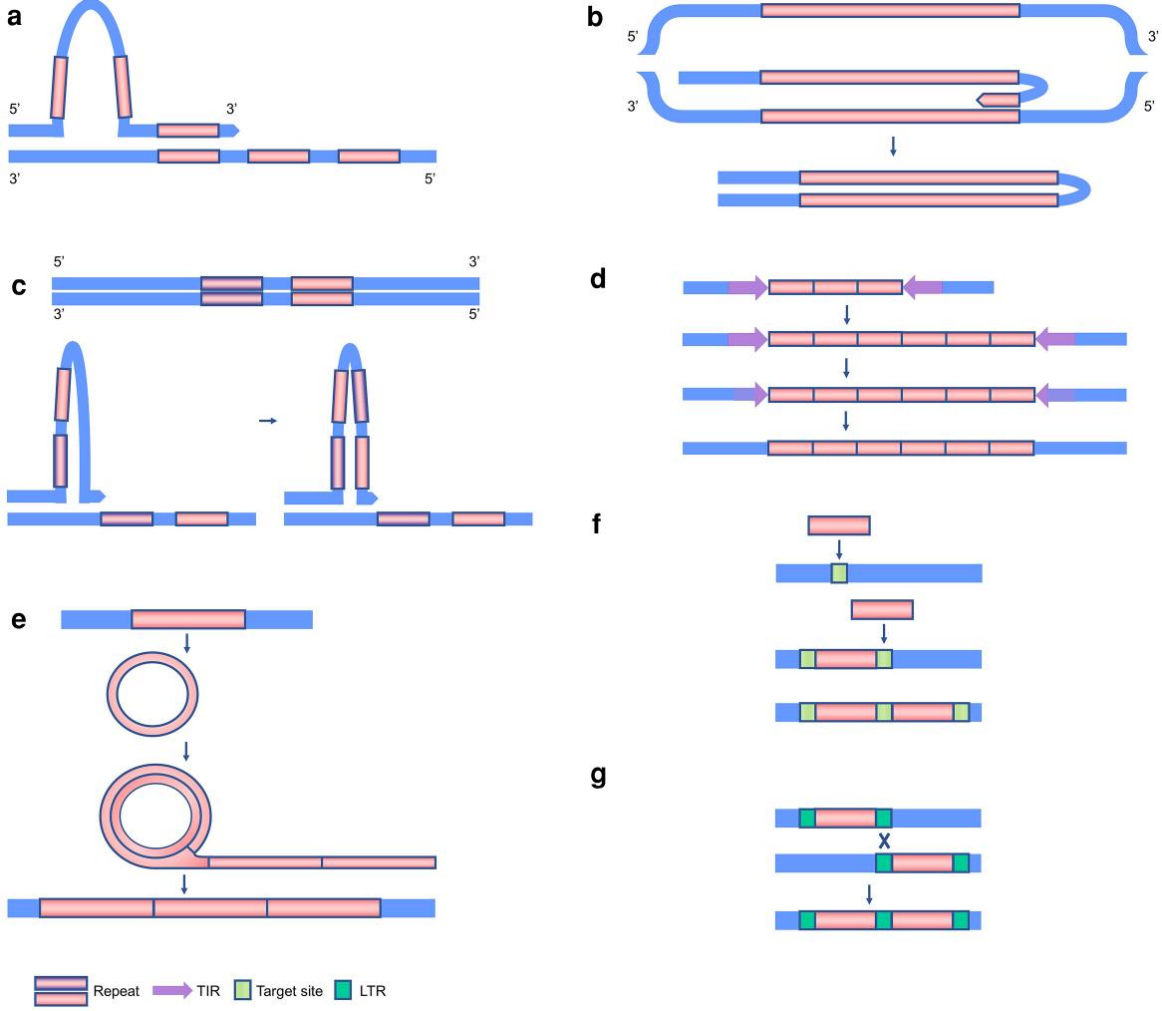
Schemes illustrating different mechanisms for the generation of satDNAs or tandem repeats. a) Staggered mispairing in the leading DNA strand results in a duplication of the template sequence. Adapted from Moran and Morish.^69^ b) Formation of the duplicated segment after newly synthesized DNA strand folds back on itself creating a loop structure. The newly formed extrachromosomal stem-and-loop structure can be incorporated into a new location in the genome. Adapted from Izsvák et al.^70^ c) Molecular mechanisms for creating tandem repeats from TEs in close proximity. Stem-and-loop structure is formed during replication by the 2 elements in close proximity. Nucleases excise the loop portion of the stem-and-loop structure, and the remaining segments are joined. Repeating this process contributes to the additional extension of the construct. Adapted from Hikosaka and Kawahara.^71^ d) The expansion of tandem repeats within MITE and formation of satDNA arrays. Tandem repeats within TEs can experience copy number changes. Subsequently, TEs containing longer tandem arrays can give rise to classical satDNA arrays after accumulating mutations in the terminal segments. Adapted from Scalvenzi and Pollet.^62^ e) RCR mechanism contributing to the formation of tandem repeats. Terminator sequence can be removed from the circular DNA template resulting in aberrant replication, followed by insertion of the resulting concatemer. Adapted from McGurk and Barbash^68^ and Xiong et al.^72^ f) Multiple insertion of the same type of TE into a single genomic site, forming a tandem. Adapted from McGurk and Barbash.^68^ g) Generation of tandem sequences via recombination between LTRs of 2 different elements. Adapted from McGurk and Barbash.^68^

Regarding the mechanisms of satDNA generation, general models of satDNA evolution suggest 2 stages in their emergence: amplification processes which generates small number of tandem repeats, followed by their expansion into longer arrays.^68^ Formation of tandem repeats can occur through different mechanisms. For example, in a process not necessarily related to TEs, such DNA replication. During replication, the displacement of the DNA strand can happen, resulting in mispairing of the complementary bases and loop formation. Staggered mispairing in the leading DNA strand results in a duplication of the template sequence^69^ (Fig. 2a). These simple repeats can expand into satDNA arrays via unequal exchange.^68^

Different hypotheses exist, providing potential explanations for the formation of satDNA sequences from TEs or from repeats present within TEs. Some of them are based on the aforementioned concept of loop formation.

Izsvák et al.^70^ propose the model explaining generation of tandem repeats from a TE, based on the construction of a stem-and-loop structure. During DNA replication, after passing the TE, the newly synthesized DNA strand can fold back on itself, creating a loop structure. Subsequently, this looped strand may disassociate from the replication complex. DNA synthesis then reinitiates at the 3′ end of the loop, using the nascent strand as a new template to replicate the TE again, and forming the stem. The duplicated segment, containing 2 copies of the TE, is then released as an extrachromosomal stem-and-loop structure (Fig. 2b). This structure can be incorporated into a new location in the genome, facilitated by local homology between the target DNA sequence and the amplified extrachromosomal fragment. The mechanism is based on studying the *Angel* MITE in zebrafish, where the ability of intrastrand base pairing of single-stranded *Angel* molecules was demonstrated in vitro.^70^

The mechanism for the formation of satDNAs from Miniature Inverted-repeat TEs (MITEs) was put forward by Hikosaka and Kawahara,^71^ based on studying Xstir sequences in the genomes of several *Xenopus* species. Three types of Xstir-related structures were observed, including MITE, tandem array, and a composite structure of MITE and tandem array. The alignment analyses revealed that tandem repeats may be derived from internal sequences of the MITE. The proposed mechanism involves the formation of a stem-and-loop structure by the 2 elements in close proximity. This structure forms during DNA replication due to the delay on 1 DNA strand. During the delay, nucleases excise the loop portion of the stem-and-loop structure, and the remaining segments are joined together. As a result, a ‘hybrid’ element is formed, containing segments from both of the original elements. Repeating this process contributes to the additional extension of the construct. The resulting sequences still represent interspersed repeats; however, with the involvement of recombination processes, they could progress into arrays of tandem repeats, satDNAs (Fig. 2c).

The mechanism proposed by Scalvenzi and Pollet^62^ explains the evolution of MITE and related satDNAs. It is based on the Tc1/mariner MITE in *Xenopus*, named miDNA4. MiDNA4 possesses a satellite DNA that exists as a single monomer or as an array of a variable number of copies. They suggest that the ancestral MITE captured a pre-existing tandem repeat. The satDNA-like sequence they described is flanked by AT-rich sequences that form short direct repeats of 5 or more base pairs. They propose that these microhomologies can lead to internal deletions and integrations during the processes of DNA replication or repair. This can result in longer internal arrays. Over time, MITEs containing longer tandem arrays can give rise to classical satDNA arrays devoid of inverted repeats at element ends, by accumulating mutations in the terminal segments (Fig. 2d).

TE Helitrons have the ability to capture various fragments of the host genome,^73^ which often include tandem repeats, as shown in multiple studies.^65,74–78^ DNA motifs that are promoting the formation of stem-and-loop structures, like direct and inverted repeats or palindromes, can also be found within the structure of Helitron/Helentron elements.^73^ They enable the Helitrons to participate in the above-described processes. In addition, Helitrons employ several mechanisms for their propagation and for the amplification or tandemization of their segments. They utilize an RCR mechanism during transposition.^72^ The RCR initiates at the 5′ end and advances towards the 3′ end, where a terminal hairpin structure serves as a recognition site for termination and subsequent DNA cleavage. Through an intramolecular recombination event involving internally repeated sequence, the 3′ terminator sequence can be removed from the circular DNA template. If that occurs, the subsequent cycle of replication generates a tandem array of truncated TEs. This causes the sequences at the Helitron’s 5′ end to become amplified more frequently than those at the 3′ end. Such formation of incomplete Helitron templates may contribute to further partial RCR, resulting in additional multiplication of the internal sequences (Fig. 2e). Finally, the tandemized, truncated Helitron copies are integrated into the new genomic location.^72^ The mechanism proposed by Xiong et al.^72^ is based on their analysis of 27 plant genomes, which revealed numerous tandem arrays of partially decayed, truncated Helitrons. Many of the detected arrays had multiple 5′ but single 3′ Helitron end, while the number of repeats in arrays ranged from several to several hundreds.

By analysing numerous genomes from 5 populations of *Drosophila melanogaster*, McGurk and Barbash^68^ revealed that TEs commonly form dimers. Their results suggest that insertion site preference is the major mechanism by which dimers are formed and that their formation is related to the periods of active transposition. They believe that the abundance of TE dimers has the potential to provide source material for expansion into satDNA arrays, based on their discovery of copy number expansion of the DNA transposon hobo to 16 tandem copies. These authors present multiple potential mechanisms for generating tandem repeats from TEs, 1 of which is the previously described RCR. The second mechanism relies on a double or multiple insertion of the same type of element into a single genomic site, forming a tandem. This is possible for TEs that create target site duplications (TSDs) upon insertion, as this allows for subsequent insertion(s) into the same target site (Fig. 2f). The third mechanism focuses on satDNAs derived from TEs containing repetitive segments such as LTRs and/or tandemly repeated regulatory motifs. These segments serve as substrates for expansion by unequal exchange (Fig. 2g). For example, ectopic recombination between LTRs of 2 different elements could generate tandemized retrotransposons that share 1 LTR.^68^ Similar mechanism was proposed by Wong and Choo,^79^ based on similarities between TE components and satDNAs reported in various organisms, such as wheat, *Arabidopsis*, *Drosophila*, and the Cetaceans. They suggested that satDNA repeats originate from the duplication of a portion of a TE sequence. This duplication occurs through unequal crossing-over between homologous TE elements, which may be located on the same chromosome or on different chromosomes, presumably in a similar way as in Fig. 2g.

Zattera and Bruschi^44^ update and summarize recorded cases of TEs that have given rise to the tandem repeat sequences. Based on that, they propose that Non-Homologous End-Joining (NHEJ) and Non-Allelic Homologous Recombination (NAHR) DNA repair mechanisms may contribute to the expansion of satDNAs from TE. If microhomology-initiated NHEJ occurs between sister chromatids, it can result in a variety of events, including sequence duplications. This mechanism is known to play a dominant role in gene duplications and is significant in completing some TE-related instability events.^44^ The NAHR mechanism offers important insights into the expansion of repeat arrays. It relies on the location of the paralogous TE copies, which serve as the template for the repair of the chromatid that has suffered the double-strand break. This may lead to tandem duplications of TEs (in the same way as depicted in Fig. 2g). This way, intra- or inter-chromatid NAHR may contribute to the expansion of the initial repeat in a way similar to the unequal crossovers.^44^

## 5. SatDNAs emerging from TE heterochromatin graveyards

As discussed above, it has previously been observed that particular satDNA could be derived from a particular TE. Here, we would like to discuss 1 specific type of composite satDNA monomers, which has not been addressed previously. In several instances, we have observed that the sequence of a satDNA monomer can contain multiple short segments that resemble various types of TEs.^54,55^ In these cases, whether the satDNAs originated from TEs was debatable as they could not be assigned to any specific TE. The origin and the characteristics of monomer sequences structured in this way are currently an open question, as they appear to result from a series of complex events. In this chapter, we propose a scenario that aims to explain their origin.

TEs are frequently enriched in constitutive heterochromatin, part of the genome with a reduced number of functional genes.^39^ TEs can insert themselves next to the other repetitive sequences via shared TSD, as described above. Additionally, they can integrate into other repetitive sequences, including previously inserted TEs of the same or different type. The insertion of TEs into other TEs produces what is known as nested insertion.^80^ Therefore, multiple insertions are common, and such loci are known to serve as hotspots and target sites for further TEs insertions.^46,81^ Sometimes, due to the imperfect process of transposition, truncated versions of TEs may be integrated,^72^ increasing the diversity of sequences at the insertion sites.

Many of the inserted copies of TEs progressively accumulate mutations and deletions over time.^82^ Genomic loci that are rich in numerous mutated and/or truncated elements in close proximity were observed.^47,55^ This can result in genomic segments that present short stretches of similarity with different TEs, and over time, their remnants eventually diverge to the point of almost being unrecognizable as TEs.^82^ Related to this, the term ‘graveyard of dead transposons’ has often been used for heterochromatin because it harbours numerous remnants from ancient TE insertions,^83^ features a ‘clustered-scrambled’ organization and a high density of repetitive sequences,^84^ and contains piRNA clusters that are particularly enriched in TE relics.^85^

We have reported the presence of the complex and ‘shuffled’ loci in the genome of the bivalve *Crassostrea gigas.*^47^ These loci appear to be generated by insertion, deletion, tandemization, and recombination events, involving satDNAs and structural components of the Helitron TE. In these instances, different parts of the elements were truncated, or tandemized, and such structures were found inserted within other (complete or truncated) elements. We found central arrays of tandem repeats within Helitron/Helentron elements that were oriented in different directions or organized in arrays of different lengths. The same type of arrays was observed both within and outside of the elements, and different types of tandem arrays were identified within a single element, as well as the same type of arrays being found across different Helitron elements.^47^

While monomer sequences of different satDNAs have very little or nothing in common, they frequently contain distinct sequence features, such as conserved motifs, inverted repeats, and palindromes.^86^ It has been proposed that these structural features may play a role in providing signals that aid mechanisms responsible for the fast proliferation of satDNA repeats, both within arrays and throughout the genome.^45^

**Fig. 3.**
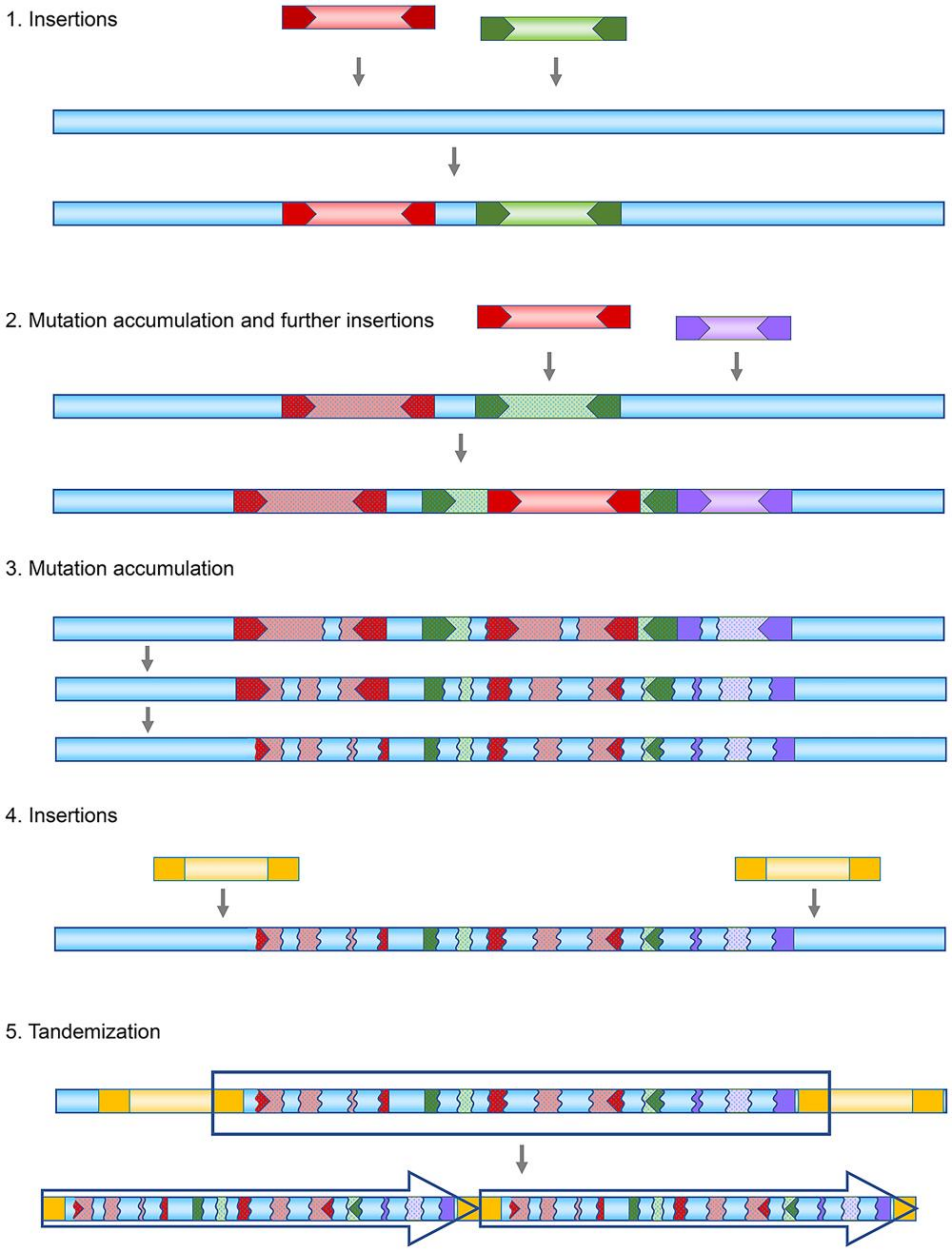
The proposed mechanism for forming composite satDNA monomers with short stretches of similarity to multiple TEs through a series of interlacing events, including insertions, mutation accumulation, and sequence degeneration.

In the genomic sites described above, DNA segment tandemization could occur through different mechanisms, aided by such sequence motifs which often accompany TEs. We propose that the tandemization and subsequent propagation of DNA segments from these genomic regions would result in novel satDNA repeats. These repeats are likely to include short stretches of similarity to different types of TEs within the monomer sequence (Fig. 3). Therefore, such satDNA sequences would indeed have their origin in what we refer to as ‘TE graveyards’. These composite satDNA sequences reveal that the DNA found in ‘graveyards’ is recycled, transforming heterochromatin TE graveyards into dynamic DNA ‘recycling yards’.

It has been proposed that degraded copies of interspersed repeats may constitute a significant fraction of unassigned genome sequences.^87^ The potential roles of these deteriorated elements as resources for host genomes have also been discussed. Truncated TE copies can modulate host gene expression by serving as new regulatory sequences, alternative splice sites, polyadenylation signals, and new transcription factor binding sites.^88^ We would add the formation of novel satDNA sequences to the potential usage of these remnants.

The considerations presented raise compelling evolutionary questions: How does selection operate on the newly generated satDNA sequences? Do the newly generated composite satDNA sequences acquire functional roles?

Whether these sequences obtain important roles in the genome and participate in functional interactions or not, this underscores a fascinating reality of genomes utilizing the available DNA with remarkable efficiency. Composite satDNA sequences show us that even the DNA residing in ‘graveyards’ is being recycled, transforming heterochromatin TE graveyards into productive ‘recycling yards’.

## 6. Conclusions and future perspectives

In this article, we have outlined the importance of satDNA sequences and TEs in genome evolution and architecture, and their involvement in various genomic processes and functions. It is also evident that TEs and satDNAs form a complex network of sequences that significantly impact the structure and, ultimately, the functionality of every eukaryotic genome. The intricate and wide-ranging connections between TEs and satDNAs show that these 2 types of sequences exist in a genome in various (and transient) forms. Here, we put forward the idea that even heavily shuffled and degraded TE remnants residing in the ‘heterochromatin graveyards’ give rise to the novel composite satDNA sequences, turning such genomic loci into DNA ‘recycling yards’.

This observation offers a foundation for various future studies that will explore the causes, mechanisms, and possible functional significance of TE-based composite repetitive components in eukaryotic genomes. Gaining insights into these aspects will deepen our understanding of the broader implications of such sequences in genome architecture and function.
